# THBS2 as a prognostic biomarker for patients diagnosed with metastatic pancreatic ductal adenocarcinoma

**DOI:** 10.18632/oncotarget.28099

**Published:** 2021-10-26

**Authors:** Phyllis A. Gimotty, Jacob E. Till, Shirsa Udgata, Naomi Takenaka, Stephanie S. Yee, Michael J. LaRiviere, Mark H. O’Hara, Kim A. Reiss, Peter O'Dwyer, Bryson W. Katona, Daniel Herman, Erica L. Carpenter, Kenneth S. Zaret

**Affiliations:** ^1^Division of Biostatistics, Department of Biostatistics, Epidemiology, and Informatics, Abramson Cancer Center, Perelman School of Medicine, University of Pennsylvania, Philadelphia, PA, USA; ^2^Division of Hematology-Oncology, Department of Medicine, Abramson Cancer Center, Perelman School of Medicine, University of Pennsylvania, Philadelphia, PA, USA; ^3^Institute for Regenerative Medicine, Department of Cell and Developmental Biology, Abramson Cancer Center, Perelman School of Medicine, University of Pennsylvania, Philadelphia, PA, USA; ^4^Department of Radiation Oncology, University of Pennsylvania Perelman School of Medicine, Philadelphia, PA, USA; ^5^Division of Gastroenterology, Department of Medicine, University of Pennsylvania Perelman School of Medicine, Philadelphia, PA, USA; ^6^Department of Pathology and Laboratory Medicine, University of Pennsylvania, Philadelphia, PA, USA; ^*^These authors contributed equally to this work

**Keywords:** pancreatic cancer, metastasis, biomarkers, prognosis, THBS2

## Abstract

Patients newly diagnosed with metastatic pancreatic ductal adenocarcinoma generally have poor survival, with heterogeneous rates of progression. Biomarkers that could predict progression and/or survival would help inform patients and providers as they make care decisions. In a previous retrospective study, we discovered that circulating thrombospondin-2 (THBS2) could, in combination with CA19-9, better distinguish patients with PDAC versus healthy controls. Here we evaluated whether THBS2 levels, previously not known to be prognostic, were associated with outcome in 68 patients at time of diagnosis of metastatic PDAC. Specifically, we interrogated the association of THBS2 level, alone or in combination with CA19-9, with progression by 90 days and/or survival to 180 days. The results indicate that elevated THBS2 levels alone, at the time of a metastatic PDAC diagnosis, can identify patients with a shorter time to death and thus help patients and providers when planning treatment.

## INTRODUCTION

Pancreatic ductal adenocarcinoma (PDAC) is often diagnosed at advanced stages of disease, with unresectable tumors that respond poorly to systemic therapy [1, 2]. The low incidence of PDAC and the absence of a known genetic or familial predisposition in most cases make it challenging to develop biomarkers with necessary sensitivity and specificity for early detection [3–5]. In addition, recent studies indicate that PDAC may progress more rapidly [6] than previously appreciated [7, 8]. Indeed, the challenges of early detection have led to the suggestion that resources should be shifted towards better predictors of progression and survival for clinical management of individuals already diagnosed with PDAC [9]. Thus, the present study is to assess blood biomarkers for discriminating between outcomes for patients with metastatic PDAC (mPDAC).

Carbohydrate antigen 19-9 (CA19-9) has been used as a diagnostic and prognostic marker for PDAC, but has insufficient power for broad utility [10]. We previously discovered candidate PDAC biomarkers with an induced pluripotent stem cell line, derived from a recurrent, late stage PDAC tumor [11]. In a retrospective study of controls and PDAC patients with various stages of disease at the time of diagnosis, plasma thrombospondin-2 (THBS2) levels, combined with serum CA19-9 [12, 13], could discriminate PDAC from controls with an overall specificity of 98% at a sensitivity of 87% [14]. Yet, in a subsequent analysis of prospectively collected samples, neither THBS2, CA19-9, nor the combination were able to sensitively predict PDAC up to one year prior to a clinical diagnosis [15].

Definitive surgical therapy is not an accepted option for mPDAC, and therefore these individuals are often offered systemic chemotherapy with a variety of potential regimens [16]. There is considerable heterogeneity in the time to disease progression and death that has been difficult to predict for each patient [17]. A biomarker that prognosticates outcome and potentially predicts treatment response for mPDAC patients would help indicate the urgency for treatment, whether to treat at all, possible stratification onto a clinical trial, and patient’s planning that inevitably accompanies a PDAC diagnosis. Given the demonstrated utility for detecting PDAC at the time of clinical presentation, here we tested THBS2 and CA19-9 at time of diagnosis as prognostic indicators for mPDAC.

## RESULTS

### Study cohort

Among the 68 study patients (Table 1), 54 (79.4%) patients had died (median = 224 days, range: 8 to 1085 days) and 14 were alive at last follow up (353 days; 206 to 1085). The 180-day mortality rate was 30.9% (21 of 68 patients). Of the 68 study patients, 62 (91%) had progression of PDAC with a median of 234 days (range: 8 to 1085); the remaining 6 patients without progression had a median follow-up time of 353 days (range: 213 to 598). The 90-day progression rate was 45.6% (31 of 68 patients). There were 18 patients who had both progression at 90 days and survived less than 180 days. One characteristic, first line therapy, differed significantly between patients alive or dead at 180 days (*p* = 0.041). There were no significant differences in age, sex, race, smoking, or diabetes status at blood draw primary location of the tumor, ECOG performance status, first line therapy or sites of metastasis between patients with and without progression at 90 days.

**Table 1:** Description of the study cohort by 180-day death and 90-day progression

	All patients		Death by 180 days		*p*-value	Progression by 90 days		*p*-value
	*n* = 68		No *n* = 47	Yes *n* = 21		No *n* = 37	Yes *n* = 31	
	*n*	%	%	%		%	%	
Age at Diagnosis					0.433			0.897
30–59	17	25.0	25.5	23.8		27.0	22.6	
60–69	29	42.7	46.8	33.3		40.5	45.2	
70–89	22	32.4	27.7	42.9		32.4	32.3	
Female	30	44.1	48.9	33.3	0.231	51.4	35.5	0.189
Caucasian	59	86.7	85.1	90.5	0.710	89.2	83.9	0.519
Smoker – Active or Former^*^	31	45.6	48.9	38.1	0.407	46.0	45.2	0.948
Diabetes – Yes or Borderline^*^	29	42.7	36.2	57.1	0.106	35.1	51.6	0.171
Primary Location					0.352			0.884
Body	14	20.6	25.5	9.5		18.9	22.6	
Body & Tail	12	17.7	19.2	14.3		13.5	22.6	
Head	20	29.4	29.8	28.6		32.4	25.8	
Tail	14	20.6	14.9	33.3		21.6	19.4	
Other	8	11.8	10.6	14.3		13.5	9.7	
ECOG Performance Status					0.278			0.617
0	20	29.4	36.2	14.3		35.1	22.6	
0–1, 1, 1.5 or 1–2	33	48.5	46.8	52.4		48.7	48.4	
2 or 3	13	19.1	14.9	28.6		13.5	25.8	
Unknown	2	2.9	2.1	4.8		2.7	3.2	
First Line Therapy					0.041			1.000
Folfirinox	25	36.8	44.7	19.1		37.8	35.5	
Gem/Abraxane	36	52.9	42.6	76.2		51.4	54.8	
Other	7	10.3	12.8	4.8		10.8	9.7	
Metastatic Sites					0.664			0.966
Liver only	36	52.9	48.9	61.9		56.8	48.4	
Lung only	7	10.3	12.8	4.8		8.1	12.9	
Other only	5	7.4	8.5	4.8		8.1	6.5	
Liver & Lung only	4	5.9	4.3	9.5		5.4	6.5	
Liver +/− Lung & Other	16	23.5	25.5	19.1		21.6	25.8	

^*^At time of blood draw.

### THBS2 and death by 180 days

**Figure 1:**
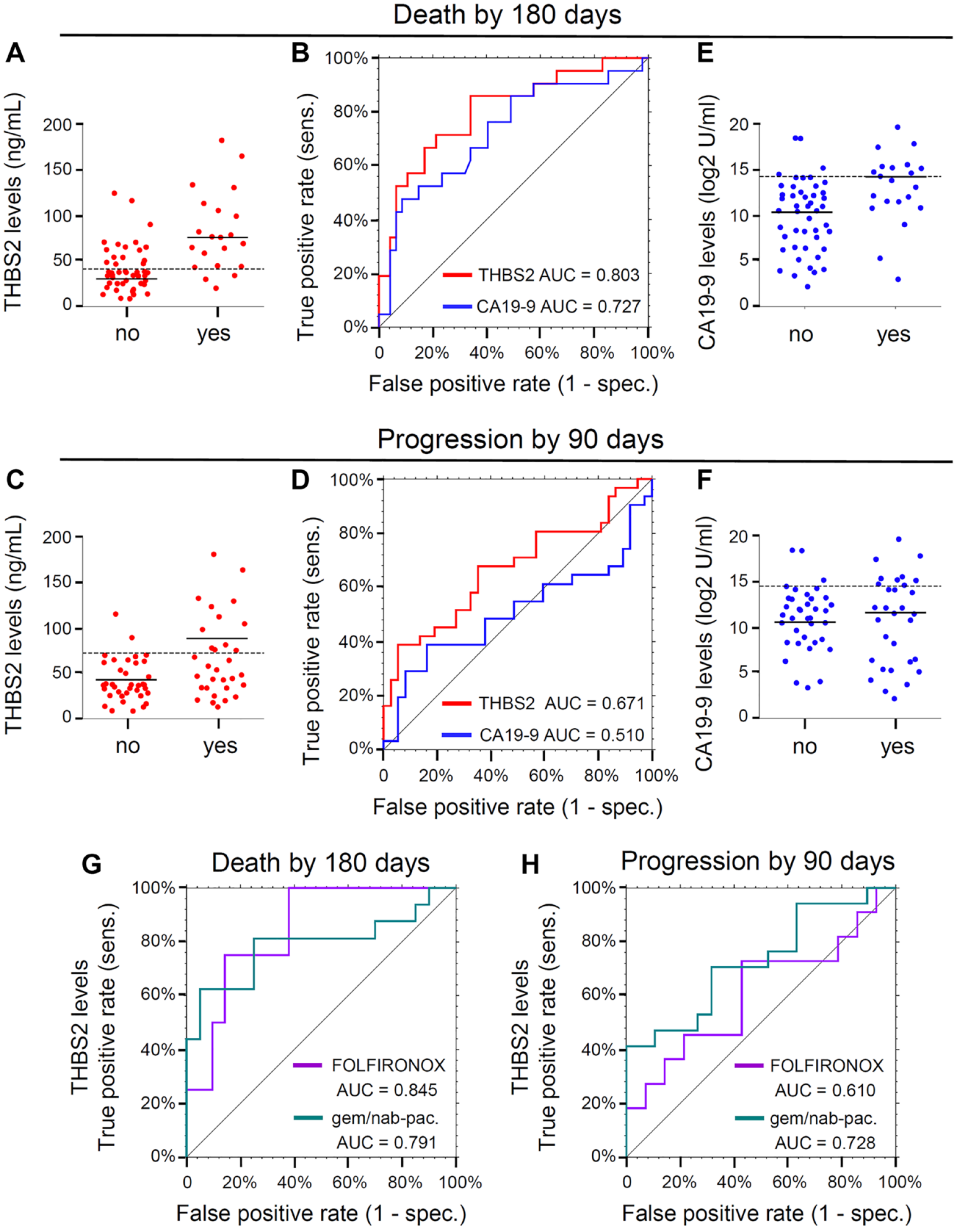
(**A**) Dot plots displaying THBS2 concentrations dichotomized by the event “Death by 180 days” (no, *n* = 47; yes, *n* = 21). Dashed line, the cut point 40.7 for THBS2 obtained by maximizing Youden’s Index. Solid lines, median values. (**B**) ROC curves for the event “Death by 180 days.” THBS2 ROC curve is represented in red (*n* = 68; AUC = 0.803, 95% CI 0.686–0.921) and CA19-9 ROC curve is represented in blue (*n* = 68, AUC = 0.727, 95% CI 0.589–0.865). “sens” is sensitivity and “spec.” is specificity. (**C**) Dot plots displaying THBS2 concentrations dichotomized by the event “Progression by 90 day” (no, *n* = 37; yes, *n* = 31). Dashed line, the cut point of 72.9 for THBS2 obtained by maximizing Youden’s Index. Solid lines, median values. (**D**) ROC curves for the event “Progression by 90 days.” THBS2 ROC curve is represented in red (*n* = 68; AUC=0.671, 95% CI 0.538–0.804) and CA19-9 ROC curve is represented in blue (*n* = 68, AUC = 0.510, 95% CI 0.362–0.658). “sens” is sensitivity and “spec.” is specificity. (**E**) Dot plots displaying Log2 CA19-9 concentrations dichotomized by the event “Death by 180 days” (no, *n* = 47; yes, *n* = 21). Solid lines, median values. Dashed line, the cut point of 1.84 for Log2CA19-9 obtained by maximizing Youden’s Index. (**F**) Dot plots displaying CA19-9 concentrations dichotomized by the event “Progression by 90 day” (no, *n* = 37; yes, *n* = 31). Solid lines, median values. Dashed line, the cut point of 2.33 for Log2CA19-9 obtained by maximizing Youden’s Index. (**G**) THBS2 ROC curves for the event “Death by 180 days” for two subgroups defined by first-line therapy: FOLFIRINOX (purple, *n* = 25, AUC = 0.845, 95% CI: 0.665–1.000), gemcitabine plus nab-paclitaxel (gem/nab-pac.) with or without HCQ (green, *n* = 36, AUC = 0.791, 95% CI: 0.623–0.958). “sens” is sensitivity and “spec.” is specificity. (**H**) THBS2 ROC curves for the event “Progression by 90 days” for two subgroups defined by first-line therapy: FOLFIRINOX (purple, *n* = 25, AUC = 0.610, 95% CI: 0.371–0.845) gemcitabine plus nab-paclitaxel (gem/nab-pac.) with or without HCQ (green, *n* = 36, AUC = 0.728, 95% CI: 0.558–0.897). “sens” is sensitivity and “spec.” is specificity.

The median THBS2 was 37 ng/ml (range: 9 to 124) for those alive at 180 days and 76 ng/ml (range: 20 to 182) for those who died (Figure 1A). The ROC derived AUC was 0.803 (95% CI: 0.696–0.921) and the interpretive threshold, balancing sensitivity and specificity, was 41 ng/ml (Figure 1A, dashed line; 1B, red line). Among the 21 patients who died within 180 days, 18 had high THBS2 (median = 77.2, range: 42.8 to 182; Figure 1A, solid line) and would be predicted to have the event (sensitivity = 85.7%). Among the 47 patients that were alive on day 180, 31 had low THBS (median = 28.5, range: 8.5 to 38.6; Figure 1A, solid line) and would be predicted not to have the event (specificity = 66%). The positive and negative predictive values were 52.9% (*n* = 34) and 91.2% (*n* = 34), respectively.

### THBS2 and progression by 90 days

Median THBS2 was 37 ng/ml (range: 9 to 116) for the 37 patients without progression by 90 days and 54 ng/ml (range: 13 to 182) for the 31 patients who progressed within 90 days (Figure 1C). The ROC derived AUC was 0.671 (95% CI: 0.538–0.804) and the cut point for THBS2 was 72.9 (Figure 1C, dashed line; 1D, red line). Among the 31 patients with progression by 90 days, 12 had high THBS2 (median = 109 ng/ml, range: 75 to 182; Figure 1C, solid line), corresponding to a sensitivity of 39%. Among the 37 patients without progression by 90 days, 35 had low THBS (36.7 ng/ml; 8.5 to 70.4; Figure 1C, solid line), corresponding to a clinical specificity of 95%. The positive and negative predictive values were 85.7% (*n* = 14) and 64.8% (*n* = 54), respectively.

### CA19-9 and death by 180 days

CA19-9 levels are routinely checked as part of clinical monitoring of patients with PDAC, including at time of initial diagnosis. The median log2-CA19-9 was 10.5 (range: 2.2 to 19) for those alive and 14 (range: 3.0 to 20) for those who died by 180 days (Figure 1E, solid lines). The ROC derived AUC was 0.727 (95% CI: 0.589–0.865) and the cut point for log2-CA19-9 was 14.2 (Figure 1E, dashed line; 1B, blue line). Among the 21 patients who died (Figure 1E), 10 had high log2-CA19-9 (median = 15.2, range: 14.2 to 19.6) and would be predicted to have the event (sensitivity = 47.6%). Among the 47 patients were alive (Figure 1E), 43 had low log2-CA19-9 (median = 10.4; range: 2.2 to 14.1), corresponding to a clinical specificity of 92%. The positive and negative predictive values were 71.4% (*n* = 14) and 79.6% (*n* = 54), respectively.

### CA19-9 and progression by 90 days

The median log2-CA19-9 was 11 (range: 3.4 to 18.4) for the 37 patients without progression by 90 days and 12 (range: 2.2 to 19.6) for the 31 patients who progressed within 90 days (Figure 1F, solid lines). The ROC derived AUC was 0.510 (95% CI: 0.362–0.658) and the cut point for log2-CA19-9 was 2.33 (Figure 1F, dashed line, 1D, blue line). Among the 31 patients who had progression by 90 days (Figure 1F), 9 had high log2-CA19-9 (median = 15, range: 15 to 20) and would be predicted to have the event (sensitivity = 29%). Among the 37 patients who did not progress in 90 days (Figure 1F), 34 had low log2-CA19-9 (11.0; 3.4 to 14.4) and would be predicted not to have the event (specificity = 92%). The positive and negative predictive values were 75% (*n* = 12) and 60.7% (*n* = 56), respectively.

### Comparison of the THBS2 and CA19-9 AUCs

The difference between AUCs for the THBS2 and CA19-9 ROC curves for death by 180 days (Figure 1B) was 0.077 (= 0.803–0.727). The AUCs of these two ROC curves were not significantly different (*p* = 0.413). The difference between the AUCs for the THBS2 and CA19-9 ROC curves for progression by 90 days (Figure 1D) was 0.161 (= 0.671–0.510). These ROC curves were significantly different (*p* = 0.049).

### Combination of THBS2 and CA19-9

Multivariable logistic regression analyses were used to evaluate whether a combination of THBS2 and log2-CA19-9 values would be a useful biomarker of death by 180 days and/or progression by 90 days. Table 2 presents the univariable and multivariable regression models. In these analyses, THBS2 and log2-CA19-9 measurements were each normalized by subtracting their respective sample means and dividing by their sample standard deviations. In the univariate models the THBS2 coefficients for both death by 180 days and progression by 90 days were statistically significant (*p* < 0.001 and *p* = 0.009, respectively). However, the log2-CA19-9 coefficient was significant only for death by 180 days (*p* = 0.008). For death by 180 days, the AUC for THBS2 alone (0.803) and the AUC for the best linear predictor from the model with THBS2 and log2-CA199 was higher (0.843), but these AUCs were not significantly different (*p* = 0.224).

**Table 2:** Univariate and multivariate logistic regression models for 180-day death and 90-day progression including THBS2 and/or Log2-CA19-9 with area under the curve for predictions

	Univariable models				Multivariable models^3^			
	Coeff	*p*-value	AUC	95% CI	Coeff	*p*-value	AUC	95% CI
**Death within 180 days**								
Intercept	---	---	---	---	−4.732	<0.001	0.843	0.745–0.941
THBS2^1^	0.036	<0.001	0.803	0.686–0.921	0.035	0.002		
Log2-CA199^1^	0.212	0.008	0.727	0.589–0.865	0.171	0.058		
**Progression within 90 days**								
Intercept	---	---	---	---	−0.648		0.708	0.583–0.833
THBS2^2^	0.023	0.009	0.671	0.538–0.804	0.026	0.005		
Log2-CA199^2^	−0.014	0.819	0.490	0.342–0.638	−0.084	0.230		

^1^The intercepts in the univariate regression models for 180-day death were −2.864 and −3.237 for THBS2 and Log2-CA19-9, respectively. ^2^The intercepts in the univariate regression models for 90-day progression were −1.386 and −0.029 for THBS2 and Log2-CA19-9, respectively. ^3^Interaction was not significant in the multivariate model for 180-day death (*p* = 0.420) or for 90-day progression (*p* = 0.106).

### THBS2 and first-line therapy

To investigate whether THBS2 differed depending on type of first-line therapy utilized, two subgroups from a clinical trial [18] were defined who received either FOLFIRINOX (*n* = 25) or gemcitabine plus nab-paclitaxel with or without hydroxychloroquine (HCQ) (*n* = 36). For death by 180 days, the overall ROC derived THBS2 AUC was 0.803 (95% CI: 0.686–0.921). The THBS2 AUC for the FOLFIRINOX subgroup was 0.845 (95% CI: 0.655–1.00, Figure 1G, purple) and for the gemcitabine plus nab-paclitaxel with or without HCQ subgroup it was 0.791 (95% CI: 0.632–0.958, Figure 1G, aqua). For progression by 90 days, the overall THBS2 AUC was 0.671 (95% CI: 0.538–0.804). The THBS2 AUCs in the two treatment subgroups were 0.610 (95% CI: 0.371–0.850, Figure 1H, purple) and 0.728, (95% CI: 0.558–0.897, Figure 1H, aqua), respectively. While patients treated with FOLFIRINOX were more likely to be alive at 180 days (84%) than those treated with gemcitabine and nab-paclitaxel (56%, Table 1), for both of the first-line therapies THBS2 was a potential prognostic biomarker for death by 180 days.

## DISCUSSION

Although considerable effort has been focused on improving the detection of PDAC at an early, potentially curable stage, most patients have metastatic disease at diagnosis. As a result of low rates of response and poor outcomes for many of these patients, therapeutic decision making is difficult and could be improved by a prognostic biomarker. Circulating GPC1-expressing exosomes, several circulating RNAs, and circulating tumor DNA have all been proposed as potential prognostics; however, none have been clinically implemented [17]. CA19-9 and CEA may be used as indicators of response [10], though neither is utilized as a baseline prognostic.

We focused on endpoints with high clinical relevance: progression by 90 days, the standard period for first radiographic monitoring of patients on therapy, and death by 180 days, the prognostic requirement for hospice care. Knowing a patient’s likelihood of such poor prognoses may change the decision-making of patients and physicians, leading to differing levels of aggressiveness in treatment, opting for experimental therapies, or forgoing treatment. Similarly, the information may help physicians set expectations for clinical course and outcomes with patients and their families. Further studies on biomarker dynamics during treatment should also be informative.

Though this study is limited by small sample size and was performed at a single institution, the results warrant further study to confirm findings, validate cut points, and demonstrate replicability. Further, inclusion of other identified prognostic biomarkers and risk factors in a multi-analyte/variable clinical model may improve utility. Additionally, research into the biology underlying the THBS2 prognostic signal may reveal targetable or other measurable differences in good and poor prognostic groups.

## MATERIALS AND METHODS

### Blood collection and processing

Blood samples were collected from therapy-naive individuals with mPDAC at diagnosis at the University of Pennsylvania Abramson Cancer Center between 2015 and 2018. This prospective nonrandomized observational study (IRB #822028) was approved by the University of Pennsylvania Institutional Review Board and all individuals who had a blood sample collected signed informed consent. The ages, sex, and other subject demographics are in Table 1. Blood was collected in K2EDTA collection tubes and plasma supernatants were isolated after each of two centrifugation steps (1,600 g × 0 min and 3,000 g × 15 min), after which it was aliquoted and banked at −80°C.

### Primary outcomes

The study focused on two clinically relevant outcomes. The first, death by 180 days, was reached if the patient died within 180-days of blood sampling. The second, progression by 90 days, was reached if the patient had clinician-defined progression (based on radiographic or clinical evidence) or died within 90-days of blood sampling.

### Biomarkers

THBS2 replicate values were determined from banked plasma using a commercial ELISA kit (Bio-Techne) as described [15]. CA19-9 was measured on banked plasma samples at the Hospital of the University of Pennsylvania Clinical Endocrinology Laboratory via the Elecsys CA19-9 Immunoassay run on a Cobas e601 platform (Roche) by electrochemiluminescence per the manufacturer’s instructions. The operator was blinded to sample identity. For statistical analyses, CA19-9 values were transformed using the binary logarithm (base 2) due to the large range of the clinical values (4.5 to 793,700).

### Statistical methods

The distributions of clinical factors at diagnosis, including age and sex, stratified by progression by 90 days and death by 180 days were compared using either Fisher’s exact test (2 categories) or an exact chi-square test (>2 categories, Table 1). The area under the receiver operating curve (AUC) was computed with its 95% confidence interval (CI) for each biomarker and outcome. Potential binary cut points for THBS2 and CA19-9 were selected by maximizing Youden’s index and were used to calculate the sensitivity/specificity, and positive/negative predictive values of the two tests. Linear logistic regression prediction models were developed for each outcome to investigate whether THBS2 and log2 CA19-9, and potentially their interaction, would be more predictive than either of the two biomarkers alone. Hosmer-Lemeshow goodness-of-fit test was used to evaluate model lack of fit. For each outcome, THBS2 AUCs and 95% CIs were computed for patients based on their first-line therapy, either FOLFIRINOX (*n* = 25) or gemcitabine plus nab-paclitaxel with or without hydroxychloroquine (*n* = 36) received as part of a clinical trial [18]). *P*-values less than 0.05 were considered statistically significant. SAS Version 9.4 and NCSS 2021 were used for statistical analyses.

## Abbreviations

PDAC: pancreatic ductal adenocarcinoma
CA19-9: Carbohydrate antigen 19-9
THBS2: thrombospondin-2
mPDAC: metastatic PDAC
ng/ml: nanograms per milliliter
CI: confidence interval
ROC: receiver operator characteristic
AUC: area under the curve
