# Correction: THBS2 as a prognostic biomarker for patients diagnosed with metastatic pancreatic ductal adenocarcinoma

**DOI:** 10.18632/oncotarget.28127

**Published:** 2022-11-02

**Authors:** Phyllis A. Gimotty, Jacob E. Till, Shirsa Udgata, Naomi Takenaka, Stephanie S. Yee, Michael J. LaRiviere, Mark H. O’Hara, Kim A. Reiss, Peter O’Dwyer, Bryson W. Katona, Daniel Herman, Erica L. Carpenter, Kenneth S. Zaret

**Affiliations:** ^1^Division of Biostatistics, Department of Biostatistics, Epidemiology, and Informatics, Abramson Cancer Center, Perelman School of Medicine, University of Pennsylvania, Philadelphia, PA, USA; ^2^Division of Hematology-Oncology, Department of Medicine, Abramson Cancer Center, Perelman School of Medicine, University of Pennsylvania, Philadelphia, PA, USA; ^3^Institute for Regenerative Medicine, Department of Cell and Developmental Biology, Abramson Cancer Center, Perelman School of Medicine, University of Pennsylvania, Philadelphia, PA, USA; ^4^Department of Radiation Oncology, University of Pennsylvania Perelman School of Medicine, Philadelphia, PA, USA; ^5^Division of Gastroenterology, Department of Medicine, University of Pennsylvania Perelman School of Medicine, Philadelphia, PA, USA; ^6^Department of Pathology and Laboratory Medicine, University of Pennsylvania, Philadelphia, PA, USA; ^*^These authors contributed equally to this work; ^**^Co-corresponding authors

**This article has been corrected:** Authors Kenneth S. Zaret and Erica L. Carpenter contributed equally as corresponding authors. The footnote to the author list indicating both individuals as co-corresponding authors was unintentionally omitted. The proper footnote is shown below:

^**^Co-corresponding authors

In addition, the paper’s title has been corrected to read, ‘THBS2 as a prognostic biomarker for patients diagnosed with metastatic pancreatic ductal adenocarcinoma.’ The authors declare that these corrections do not change the results or conclusions of this paper.

Original article: Oncotarget. 2021; 12:2266–2272. 2266-2272. https://doi.org/10.18632/oncotarget.28099

